# Studies on Chemical Composition, Structure and Potential Applications of *Keratoisis* Corals

**DOI:** 10.3390/ijms24098355

**Published:** 2023-05-06

**Authors:** Mieczysław Gorzelak, Dorota Nowak, Andrzej Kuczumow, Dianne M. Tracey, Witold Adamowski, Jakub Nowak, Jakub Kosiński, Jacek Gągała, Tomasz Blicharski, Agnieszka Lasota, Mirosław Jabłoński, Jarosław Pawlicz, Maciej Jarzębski

**Affiliations:** 1 Department of Orthopedy and Rehabilitation, Medical University of Lublin, 20-059 Lublin, Poland; 2 Lab 196, Radawiec Duży 196, 21-030 Motycz, Polandandrzej.kuczumow@gmail.com (A.K.);; 3 National Institute of Water and Atmospheric Research Ltd., Wellington 6022, New Zealand; 4 Department of Environmental Engineering, Bydgoszcz University of Science and Technology, 85-796 Bydgoszcz, Poland; 5 Department of Orthopedics and Traumatology, Medical University of Lublin, K. Jaczewskiego 8, 20-090 Lublin, Poland; 6 Chair and Department of Jaw Orthopedics, Medical University of Lublin, Chodźki 6, 20-093 Lublin, Poland; 7 Department of Orthopedics and Traumatology, Poznan University of Medical Sciences, 28 Czerwca 1956 135/147, 61-545 Poznań, Poland; 8 Department of Physics and Biophysics, Poznań University of Life Science, 60-637 Poznań, Poland

**Keywords:** bamboo corals, *Keratoisis*, gorgonin, keratin, bromine, annual growth rings

## Abstract

The chemical composition and structure of bamboo octocoral *Keratoisis* spp. skeletons were investigated by using: Scanning Electron Microscopy SEM, Raman Microscopy, X-ray Diffraction XRD, Laser Ablation–Inductively Coupled Plasma LA-ICP, and amino acid analyzers. Elements discovered in the nodes (mainly organic parts of the skeleton) of bamboo corals showed a very interesting arrangement in the growth ring areas, most probably enabling the application of bamboo corals as palaeochronometers and palaeothermometers. LA-ICP results showed that these gorgonian corals had an unusually large content of bromine, larger than any other organism yet studied. The local concentration of bromine in the organic part of the growth rings of one of the studied corals grew up to 29,000 ppm of bromine. That is over 440 times more than is contained in marine water and 35 times more than *Murex* contains, the species which was used to make Tyrian purple in ancient times. The organic matter of corals is called gorgonin, the specific substance that both from the XRD and Raman studies seem to be very similar to the reptile and bird keratins and less similar to the mammalian keratins. The missing cross-linking by S-S bridges, absence of aromatic rings, and significant participation of β-turn organization of peptides differs gorgonin from keratins. Perhaps, the gorgonin belongs to the affined but still different substances concerning reptile and bird keratin and in relation to the more advanced version—the mammalian one. Chemical components of bamboo corals seem to have great medical potential, with the internodes as material substituting the hard tissues and the nodes as the components of medicines.

## 1. Introduction

Bamboo corals (Phylum: Cnidaria; Class: Anthozoa, Subclass: Octocorallia, Order: Alcyonacea, Suborder: Holaxonia, Family Isididae) are an abundant and widespread group of gorgonian octocorals with over 150 species recorded globally. Currently, the classification status of the family and sub-family for this key group is under revision (e.g., see Heestadt et al., 2021). Here we refer to the study species (*Keratoisis*) as Family Keratoisididae [1,2,3].

In the last few decades, there has been global interest in the taxonomy, chemical structure, and spatial distribution of deep-sea corals [4]. Recent research has focused on disturbance from fishing, mining, and mineral exploration [5,6], as well as from climate change and ocean acidification (OA) impacts due to the shifts in temperatures and salinities within which they survive [7,8,9,10,11]. Concomitant with this, interest in the Idsidids has also evolved, but little research has focused on the chemical microstructure of this group.

Contrary to the common opinion, the great majority of all coral groups belong to deep-water species. In general, most bamboo corals are located in waters below 800 m [12], with the deepest specimens recorded at 4851 m [13]. Bamboo corals are common in the Indo-Pacific, South-West Pacific, and Atlantic Oceans and are much more geographically widespread than the commonly occurring tropical zooxanthellae counterparts. Colonies can vary in shape and size; they can be tree-like and branched, whip-like, bushy, or have a candelabra form, and can range in size from small fragile colonies to robust large colonies over a few meters in height. Their polyps are non-retractile. Some bamboo corals form large underwater meadows or forests [14] populated by individuals up to 10 m high and with bases as wide as 3 m. Such environments support many invertebrates and fish providing habitat, shelter, and refuge. Living bamboo coral colonies are probably the most important ecosystem in oxygen-deficient areas [15,16]. Moreover, the fossil specimens and colonies are encountered as well [17], testifying to the very ancient origin of those species. The live and dead colonies provide useful paleo-climate records (ref). Like several other corals, bamboo corals stabilize themselves by cementation to a hard substrate (rocks, edges of seamounts and canyons, coral reef) [18]. In general, bamboo corals belong to insufficiently known organisms among all coral species. 

Keratoisidid bamboo corals produce a skeleton that is mostly branched. Each branch is composed of heavily calcified internodes and dark nodes composed of proteinaceous gorgonin with the addition of polysaccharides [19,20]. The sequence of consecutive light inorganic and dark organic parts along the length of the branch gives the organisms an impressive bamboo-like appearance. On the transverse cross-section of nodes, the simple ring or zone structure, as seen in tree rings, is apparent. The rings grow slowly with radial growth rates in the range of either 50–150 μm/year [21,22] or 130–290 μm/year [9], with a vertical growth rate at least an order of magnitude faster [17]. In parallel with the slow-growing nature of this group, bamboo corals, like several other deep-water corals, have high longevity [23].

Bamboo corals contain high-magnesium calcite and organic gorgonin. Gorgonin is a substance assumed to be very close in chemical composition to collagen [24], the latter known as an organic component of bone and dentin. The arrangement of the gorgonian is that the fibers of proteins are submerged in a proteinaceous matrix [25]. The fibers were determined by Goldberg [20] as collagen components, cross-linked with aromatic compounds, perhaps tyrosine- or thyroxine-like. In axial skeletons, the collagenous substance is enriched in tyrosine [26]. Tyrosine most likely increases the degree of cross-linking or sclerotization [18]. Goldberg [26] observed that increased cross-linking (i.e., greater tyrosine content) deepened the dark color of the organic components of corals. The color of gorgonin varies from brown to black in most Keratoisidids.

Another and more common opinion is that gorgonin is related to keratin [27,28]. Gorgonin in Keratoisids is an iodinated [29] substance involving 3,5-diiodotyrosine and even more brominated material. The role of iodine is better known [30], while the presence of bromine has been obscure until now. Still, tyrosine is very susceptible to bromine attack and further transformations [31]. The recently published paper by McCall et al. [32] proves that Br is essential for sulfilimine formation, the substance cross-linking fibers in collagen IV scaffolds of basement membranes. Another study [33] testifies that bromine compounds protect organisms from parasites. The studies on the way of formation of gorgonin lead probably to an understanding of how collagen-like (or keratin-like) materials can be synthesized under low temperature and pressure environments, characteristic of bamboo coral habitats [34]. Sherwood amino acid analyses for *Primnoa resedaeformis* (a primnoid gorgonian octocoral species) indicated that alanine (Ala: 0.72–1.71 μmol/mg); aspartic acid (Asp: 0.68–1.49 μmol/mg); glycine (Gly: 0.56–1.36 μmol/mg); serine (Ser: 0.45–1.04 μmol/mg) were the main amino acid components in gorgonin [35] of living corals and it did not change very much in fossils. The composition was rather distant from the composition of classic collagen. In addition, the results of Sherwood [29] testify to the durability of organic matter in bamboo corals, at least at the Holocene scale of time.

Goldberg [20] carried out chemical analyses on the skeletal composition of three gorgonian octocoral species. Protein content ranged from 70.1 to 91.4%, ash from 9.6 to 19.4%, lipids from 0 to 8.4%, carbohydrates from 1.24 to 3.48%, and halogens from 4.2 to 12.04% of the dried skeletal weight [36].

Calcite has been shown to be the second most important component of Keratoisidid corals [37]. The calcite content can be analyzed to help reveal climatic changes in a deep ocean environment [38]. There are a few other sources of elemental proxies that can be used to inform the ocean environment. The presence of magnesium (Mg) and sulfur (S) in the calcite matter is probably the most promising indicator of ocean temperature, and is used to indicate ocean pH [39] and composition of water [40] data. Strontium (Sr) and phosphorous (P) are not seen as useful by some [41,42]. Roark et al. [21] and Heikoop et al. [43], however, expressed a different opinion about the role of Sr as a geothermometer in bamboo corals.

Variations in ocean temperature and depth seem to influence ring growth. Hill et al. [44] claimed that Sr/Ca ratio in bamboo corals preserves the original ratio of those cation molecules in the water column. An interesting study on the role of Na and S can be found in Floeter [45]. Nevertheless, the detailed studies are ambiguous—some data testify to the fact that the rings have an annual nature, while longer-term scrutiny contradicts this conclusion [46,47]. There are potential factors, such as environmental disturbance in a particular year or lunar cycles, that could affect the coral growth zone deposition and help explain the variation in a coral’s chemical composition. A shorter lunar cycle seems to be a very important factor for corals [21,48,49], and it can cause the annual cycles to obscure. Similarly, isotopic studies, particularly those that use the ^15^N isotope, showed that decadal and not annual time resolution is better mirrored in bamboo corals [50]. Isotopic studies are probably the most useful as bioarchives in explaining reconstruction variations for past ocean conditions [51,52].

Nucleation sites occur along the gorgonin proteinaceous fibers. From these calcification centers, spherulitic crystal growth would initially occur in all directions, but as interference is encountered in most directions, the growth is restricted. The organic matrix appears to constrain the outward growth of the needle crystals, generating loculi with a finite selection of shapes.

The building material of bamboo corals attracted the attention of scientists trying to find the optimal material for bone reconstruction [53]. In this aspect, the efforts to cultivate the bamboo corals through biotechnological aquaculture were performed.

The key aims of this manuscript are to further investigate both the inorganic and organic make-up of the Keratoisidids skeleton—of both the dark gorgonin internodes, as well as the calcium carbonate nodes. Additionally, the microscale chemical structure is presented. Based on the results of this study, we highlight the potential application of the results in areas of, for example, medical research: orthopedics, stomatology, and neurology.

## 2. Results

**Figure 1:**
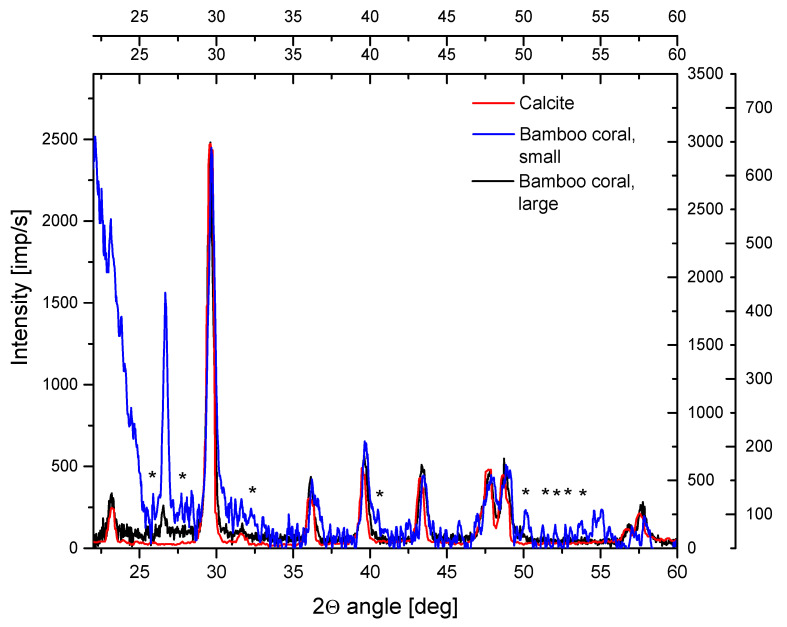
X-ray diffraction spectrum for inorganic part of small coal (blue line) and large coral (black) as compared with the spectrum of pure calcite (red), range 22–60°. Asterisks show the peaks attributed to hydroxyapatite.

We identified the inorganic part of bamboo coral as the high-magnesium calcite. It was very clearly observed in the X-ray diffraction spectrum (Figure 1). Some traces of hydroxyapatite [54] are observed in the samples of small bamboo coral, as evidenced in Figure 1. All the peaks attributed to the hydroxyapatite are greater than 3 standard deviations of the background value.

**Figure 2:**
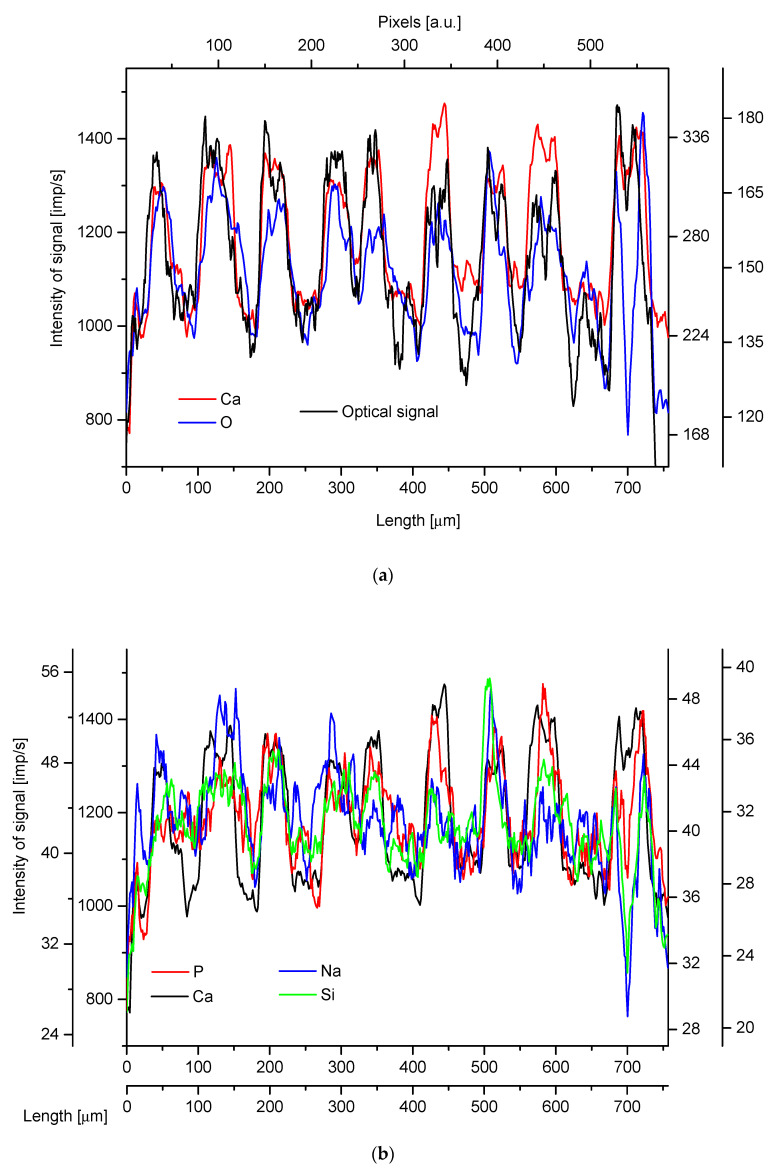
(**a**) Longitudinal profiles along the transverse cross-section of the large bamboo coral, organic node zone: optical profile—black; Ca—red; O—blue line; (**b**) comparison of different elemental profiles: P—red; Ca—black; Na—blue; Si—green line.

The part of the coral is composed of calcite mixed with organic matter in a ring-like structure. The analyses of the growth rings inside the organic nodes along the transverse cross-sections gave an impressive sequence of the consecutive layers. In Figure 2, the essential components of the calcite (Ca, O—analyzed by electron microprobe) are superimposed on the optical profile of the rings, as extracted from the grayscale levels of the sample photo. Moreover, a number of other components are strictly coupled inside the studied space with the calcite matrix. It is shown in Figure 2b, and the elements of interest are as follows: P, Ca (it further suggests the presence of hydroxyapatite), Si and Na. The remaining detected elements: Mg, Br, Cl, and S are decoupled with the white rings and are probably inside the organic matrix, but they are also somewhat shifted from one another. It testifies to the nonuniform structure of the organic layers.

**Figure 3:**
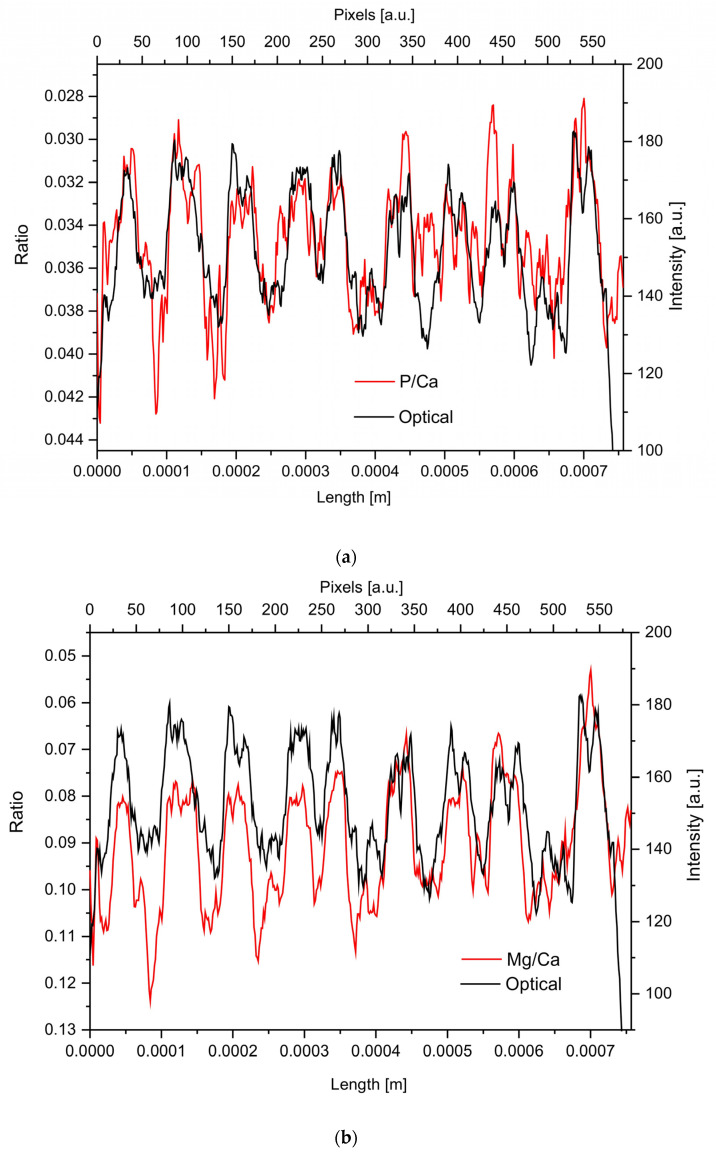

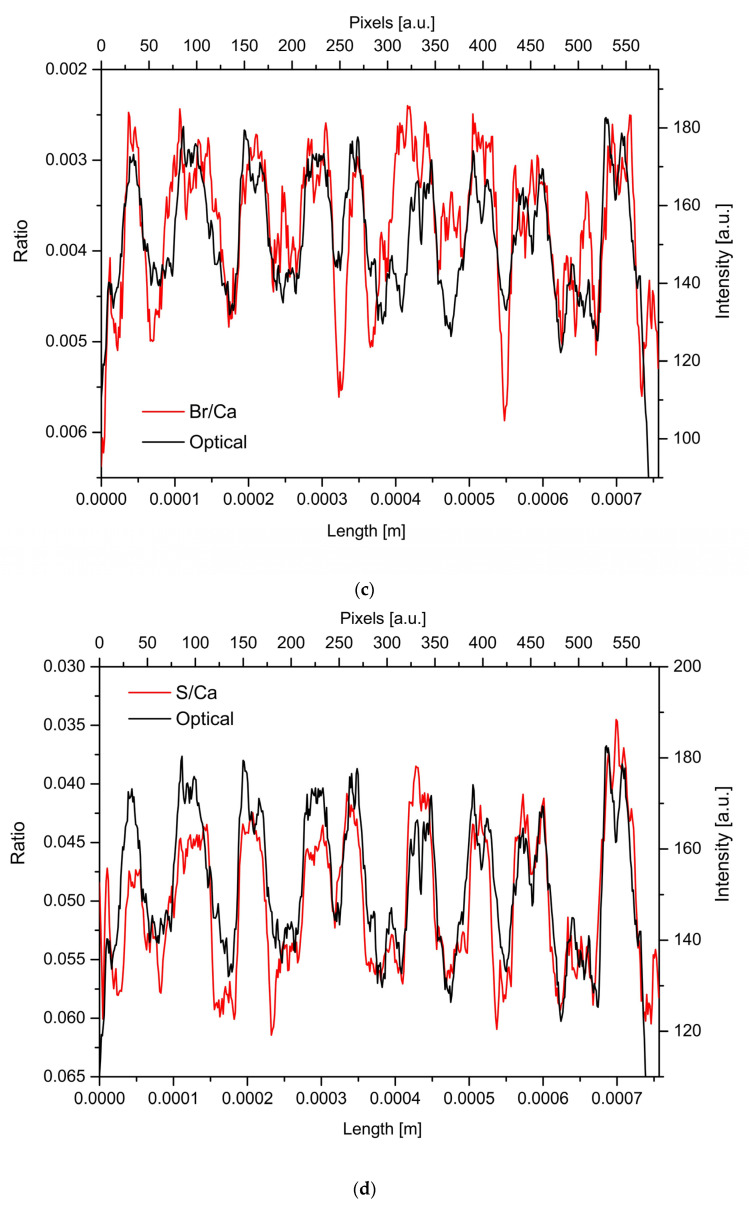
Comparison of concentrations profiles of some elements normalized against Ca contents with the optical profile: (**a**) P/Ca; (**b**) Mg/Ca; (**c**) Br/Ca; (**d**) S/Ca ratio.

The concentrations of the mentioned elements can be related to the concentration of the main component, Ca, and then the ratios can be conveniently compared with the optical profile. Some of such relative profiles (Mg/Ca, Br/Ca, S/Ca) reflect the optical profile in a very accurate way (Figure 3). In particular, the resemblance is extraordinary in the cases of P/Ca and Br/Ca ratios. It is interesting that the mentioned elements, except P, belong to those mentioned earlier as being decoupled with the optical signal. The strict coupling of relative concentrations can even suggest that calcium is joined with phosphates, bromides, or bromates and sulfates, of course in amounts corresponding to the concentrations of minor anion-forming elements. Once more, we can claim that the apatite is admixed to the calcite matrix. The results suggest in addition that sulfur is involved rather in sulfate species than in sulfur-bearing amino acids.

Due to the semiquantitative character of electron microprobe determinations and to have a more accurate idea about the contents of the elements in the bamboo corals, the point determinations made by the LA-ICP for external and internal layers in nodes of greater and smaller bamboo coral are presented in Table 1. For alkaline and alkaline earth elements (Na, Mg, Sr), the values are strongly increased in external locations. For Na, it is totally different from the situation observed by Floeter et al. [45]. For Fe and Zn, the discrepancies are variable. P is concentrated rather inside the ring system; thus, the apatite inclusions should be looked for inside nodes. The estimations for J and especially Br are strikingly great, with concentrations being clearly maximal in internal layers. The values for Br are uncommon for any other known organism. In general, the traces of foreign elements are much greater in smaller coral, while Br and J are more populated in large coral. The Mg contents can suggest that for small coral, the calcite matrix is composed of high-magnesium calcite, while for large coral, it is from normal, relatively pure calcite.

The observation of ring diameters from our measurements, oscillating around 80 μm, coincides well with the data from Roark et al. [21] and Andrews et al. [22], attributed to yearly rings. Although some internal structure inside particular annual rings is observed, it would be difficult to attribute this to lunar or decadal cycles, as suggested by some researchers. Using these profiles, the simplest chronology can be deciphered, assuming that the rings are year-long structures.

The organic contents of the bamboo corals were analyzed with reverse-phase high-performance liquid chromatography. The results are presented in Table 2 for the external and internal layers of each coral branch, respectively, and compared with similar investigations by Sherwood et al. [35]. If we compare the five most abundant amino acids in our and Sherwood’s measurements, we find alanine, glycine, serine, and arginine among them, and the results are similar in that sense. We found abundant proline, which is absent in Sherwood’s studies but was observed by Ehrlich [55], who at the same time hardly observed glycine, very abundant in our and Sherwood’s studies. Since different bamboo corals were investigated in the mentioned studies by different authors, we suppose that the amino acid compositions differ a little from the assumption, from sample to sample. We emphasized the amino acids, routinely attributed to some forms of peptide conformations in colors, which will be considered in the further text.

**Figure 4:**
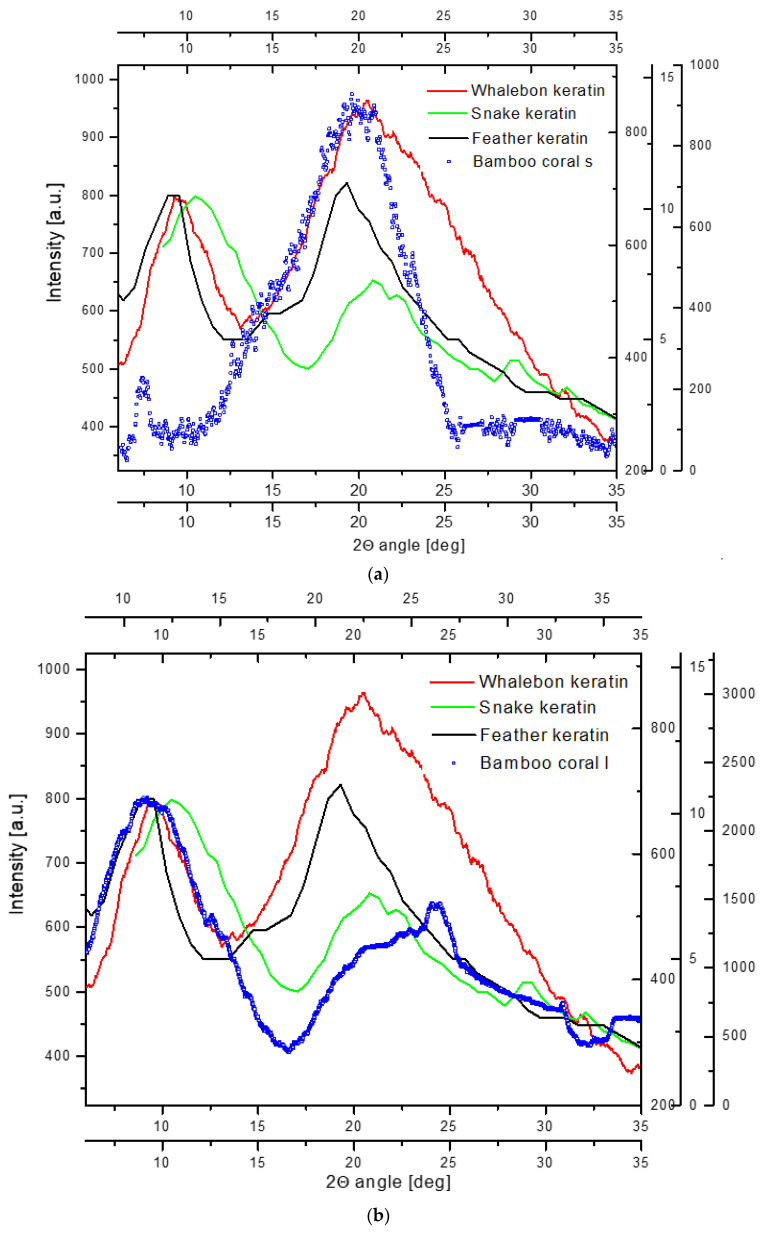

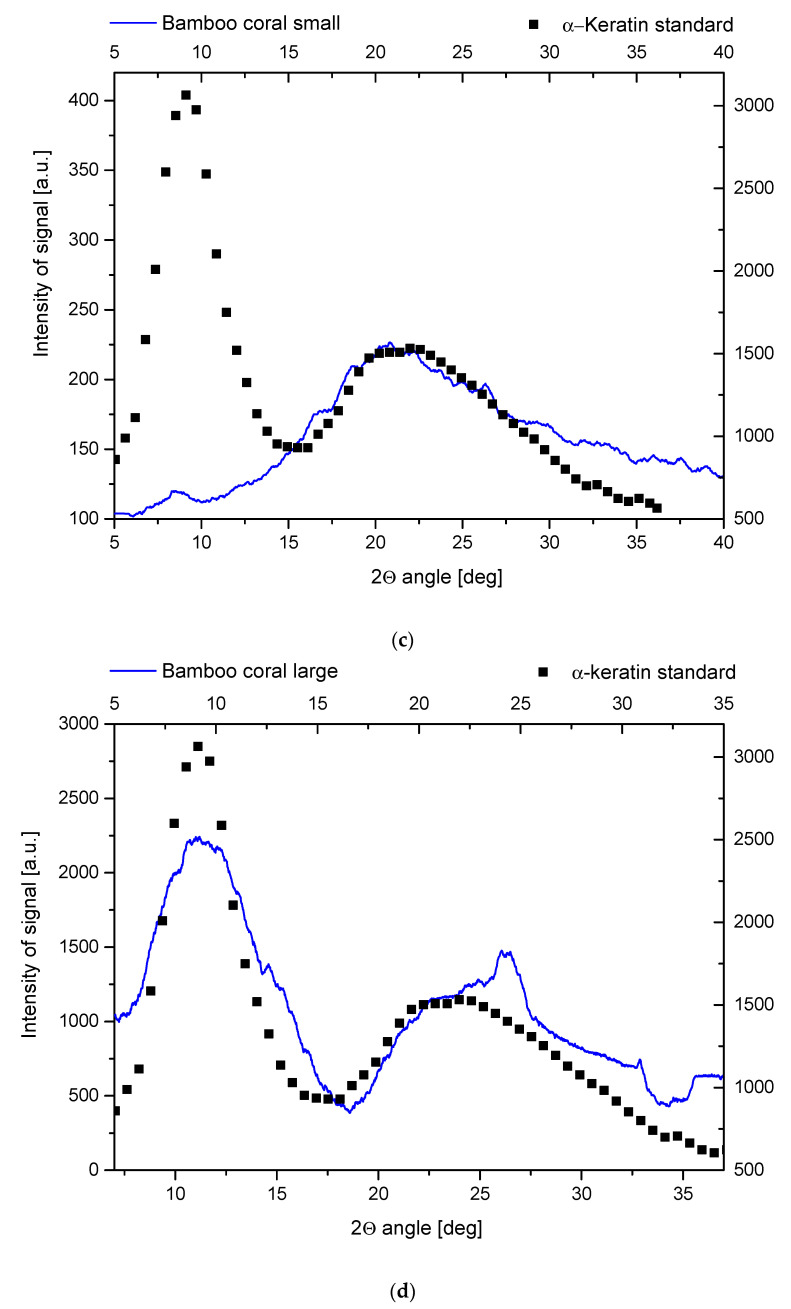
(**a**) Diffraction spectrum of the keratine of whalebone, feather, and snake compared with the spectrum of gorgonin in small coral; (**b**) the same data compared with the spectrum of gorgonin in large coral, the shift by 2°; (**c**) diffraction spectrum along the equatorial space for standard keratin, adopted from Kreplak et al. [56], compared with the spectrum of gorgonin in small coral; (**d**) the same for the keratin in large coral (shifted by 2°).

Since there are some reasons to consider gorgonin as either an analog or substance similar to or the precursor of the well-known keratins, we decided to make the comparison of the scattering patterns in the X-ray diffraction analysis of the gorgonin from small and large samples of corals with the patterns of some typical keratin samples. In Figure 4, we compared the X-ray diffraction spectra for whalebone (mammal), feather (bird), and snake (reptile) keratins with the gorgonin from small and large corals, respectively. Next, the coral gorgonians were compared with the standard α-keratin spectrum adopted from Kreplak et al. [56]. Figure 4 confirms the hypothesis about the similarity of the spatial arrangement of peptides in keratins and gorgonians: they have the same scattering components. However, the spectrum details for the large coral sample are shifted by 2 degrees.

**Figure 5:**
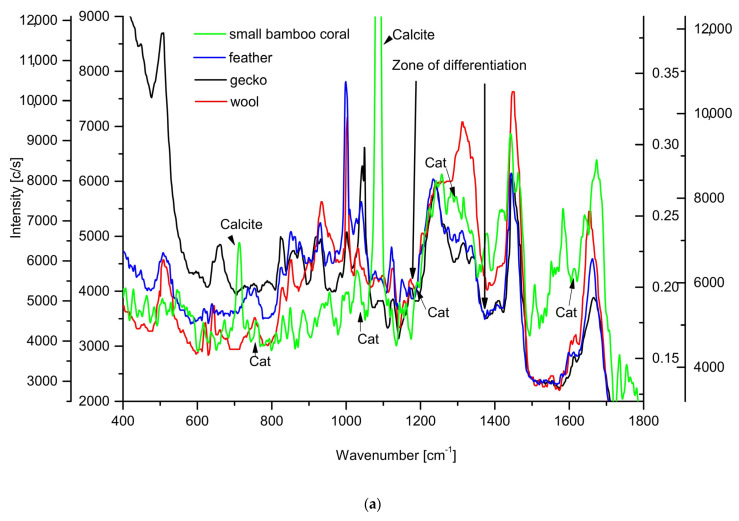

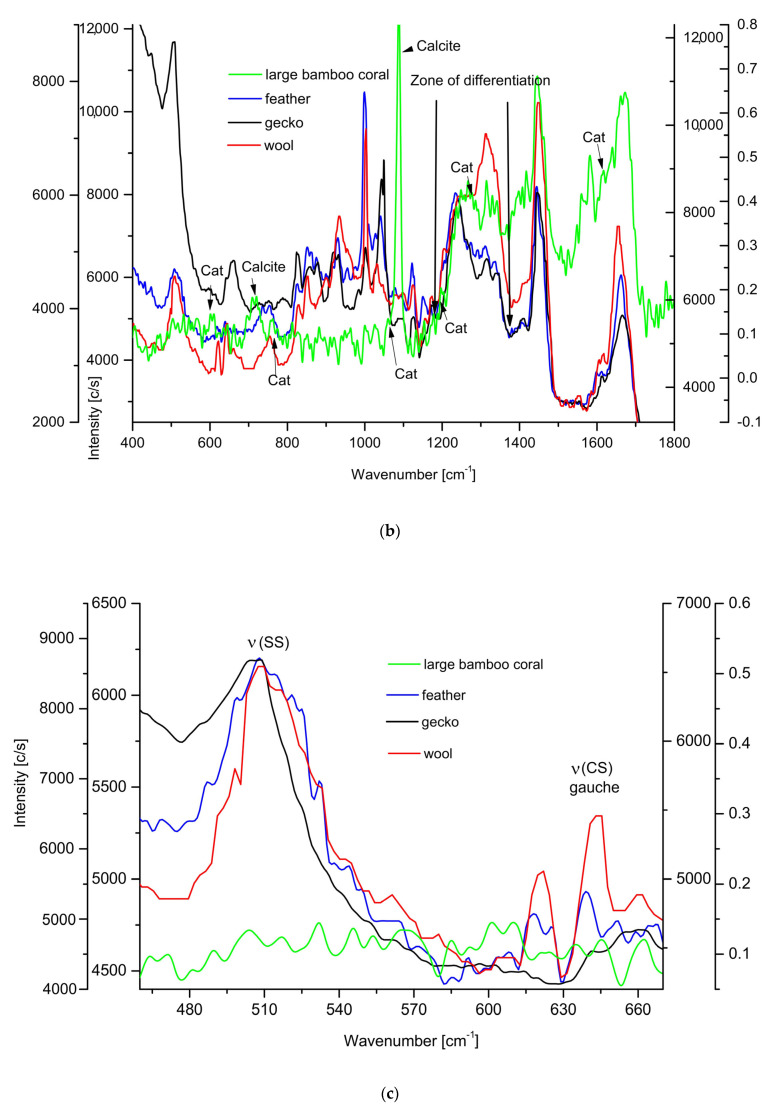

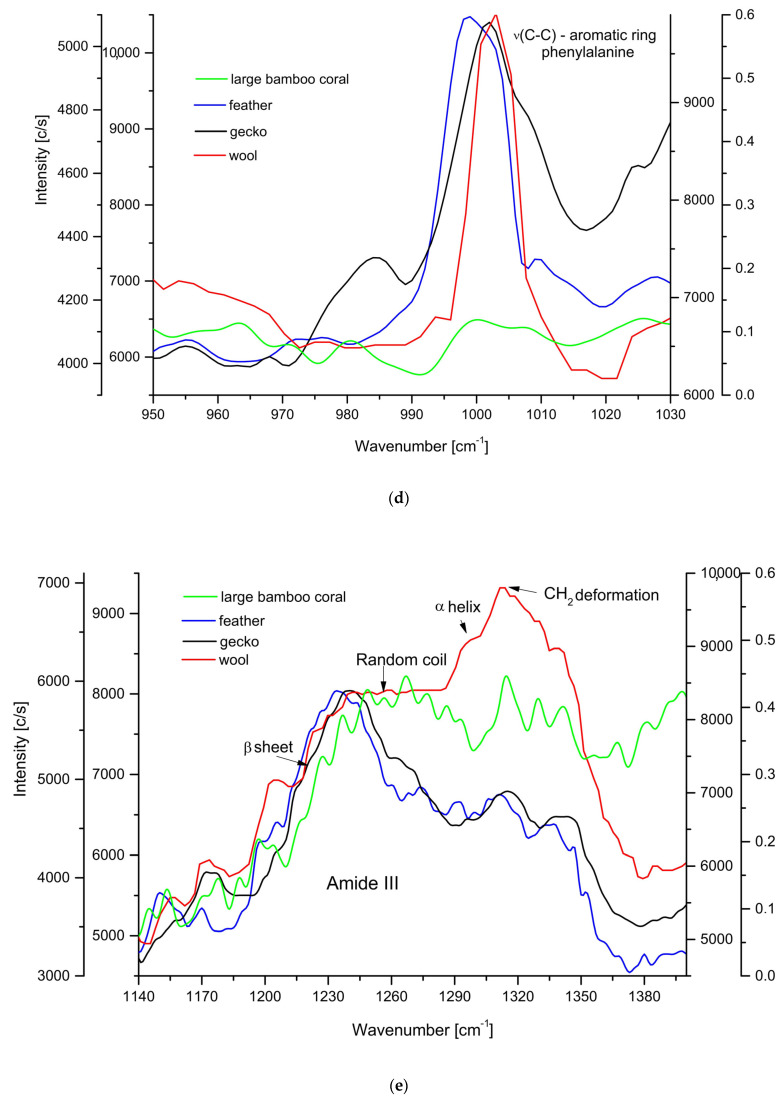

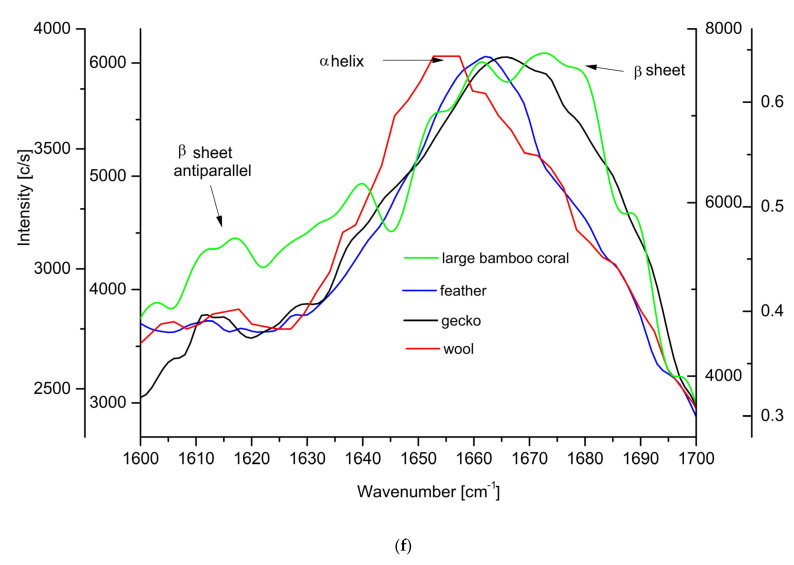
(**a**) Raman spectrum of small bamboo coral as compared with the spectra of keratin in feather, wool, and gecko, adapted from Rizzo et al. [57]; (**b**) the same for a spectrum of larger bamboo coral. The range of spectra (**a**,**b**) is from 400 to 1800 cm^−1^, to couple with the Rizzo data. The designations mean calcite—the fragments of calcite origin; Cat—the peaks which might be assigned to dicatechol cross-linking fragments [60]. Selected fragments from Raman spectra, all compared to Rizzo standards: (**c**) zone of 460 to 675 cm^−1^, showing characteristic differences between keratins and large bamboo coral for sulfur-based cross-linking; (**d**) comparison of 1003 cm^−1^ line attributed to the aromatic ring for standards, with the intensity of the line for large bamboo coral; (**e**) expanded zone of 1140 to 1390 cm^−1^ that covers amide III and CH_2_ deformations, showing characteristic differences between mammalian and bird and reptile keratins (standards adapted from Rizzo et al. [57]), as compared with small bamboo coral organic materials; (**f**) zone in the amide I range from 1600 to 1700 cm^−1^. The spectra (**c**,**d**) are normalized against strong δCH_2_ scissoring oscillation (1449 cm^−1^). The spectrum (**e**) is normalized against δCH_2_) wagging and ν(CN) in the amide III zone (1243 cm^−1^).

The Raman measurements were performed separately for the organic matter from small and large bamboo corals (Figure 5a) and compared with the set of measurements adapted from Rizzo et al. [57], who tried to prove that the keratin of reptilian or bird origin differs from mammalian ones. The similarities of gorgonin material from bamboo corals to the reptilian and bird keratin are nice, although with clear differences in some spectral regions: around 507 cm^−1^ S-S lines; 1003 cm^−1^, the aromatic ring mainly attributed to phenylalanine; 1140–1390 cm^−1^ in amide III zone; 1600–1700 cm^−1^ in the amide I and 1750 cm^−1^ band (Figure 5b,c). The comparison was also made with wide keratotic materials studies carried out by the group of Edwards [58,59]. Undoubtedly, the spectra of both bamboo coral materials are more similar to the spectrum of gecko and feather than to the spectra of mammalian keratins. Observe, please, that for the amide III zone, the bamboo coral keratin-like material lies between mammal and bird and reptile keratin. In addition, the sequence of amide I zones shows some hierarchy of materials, with coralline material somewhat outside the rest of the materials. Figure 5d confirms that phenylalanine is scarcer in large bamboo corals than in small corals and in typical keratins, which earlier resulted from the chromatographic measurements (Table II).

**Figure 6:**
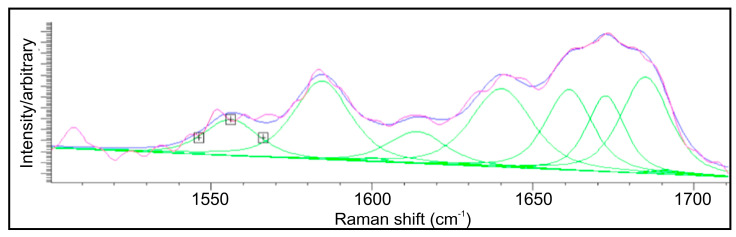
Deconvolution of amide I zone of small bamboo coral. One should see 8 separate bands; however, 7 of them are clearly visible, the 1605 cm^−1^ zone is hardly observed, and the 1698 cm^−1^ zone is absent (violet line—measured spectra, blue line—envelope of spectra, green lines—deconvoluted spectra).

The amide I zone was deconvoluted on the nine component bands, in the same way as it was performed in Skieresz-Szewczyk et al. paper [61]. The separate bands were attributed to (Figure 6): 1—1550 cm^−1^ − δ(NH), ν(CN); 2—1585 cm^−1^ − ν(CC) olefinic; 3—1605 cm^−1^ − phenylalanine/tyrosine; 4—1615 cm^−1^ − tyrosine/tryptophane; 5—1638 cm^−1^ − random coil; 6—1652 cm^−1^ − α helix; 7—1669 cm^−1^ − β sheet; 8—1685 cm^−1^ − β turn; 9—1698 cm^−1^ − β turn.

In Figure 6, the β turn component (1685 cm^−1^) is most pronounced but 1585 cm^−1^ (olefinic C-C), 1638 cm^−1^ (random coil), 1652 cm^−1^ (α helix) and 1669 cm^−1^ (β sheet) spectral entities are all moderately intensive. Lines 1550 cm^−1^ and 1615 cm^−1^ are distinguished, but their intensity is rather low.

## 3. Discussion

### 3.1. Ring Structure within Nodes

The inorganic part of bamboo corals is essentially composed of high-magnesium calcite (although, for large coral—calcite), but traces of silicon and phosphorus can also be detected, the latter confirmed in analyses by LA-ICP. The spots with elevated concentrations of the elements can be some precursors of silica and apatites [54,62], and perhaps this explains the proven ability of calcite from bamboo corals to be used as a prosthetic material for bone repair. Nevertheless, at least some part of the phosphorus should also be of organic origin. If Si (in silica) and P (in apatite) are indeed involved in an inorganic matrix, their content is below 2%, the commonly accepted detection limit for X-ray diffraction determinations; indeed, the P level is of an order of hundreds of ppm. However, the strongest lines of apatite seem to be detectable in the XRD spectrum (Figure 1).

The profiles of the ratios of different minor elements to Ca as detected by the electron microprobe show very interesting characteristics. Namely, the inverted ratios compared with the optical grayscale profiles show an extremely good similarity. It concerns the ratios of Mg/Ca, P/Ca, Si/Ca, Br/Ca, and S/Ca. In particular, the ratios of Br/Ca, S/Ca, and P/Ca are very accurate in relation to the optical profile. No single element profile is so strictly related to the optical profile. It can in an indirect way testify to the fact that the involvement of the elements mentioned is temperature-related. It is known that such optical-related elemental ratio profiles are used as geochronometers and geothermometers [63,64]. Most frequently, magnesium fulfills such a role in calcite materials of geochemical and biogeochemical origin. This time, Mg is not the most representative element in the mentioned aspect, since it does not follow the optical profile in the same strict way as, e.g., Br.

The most striking feature of the material from the bamboo coral is the extremely high or elevated concentrations of bromine in the whole material. Point analyses of corals gave results up to 2.8%, the value never met in any living organism [65]. The levels of iodine are much lower—they are about 2 orders of magnitude smaller than those of bromine. However, it is difficult to reveal the Raman C-Br lines due to their rather very weak intensity, even with the help of the earlier determined line assignations [66,67,68]. The very similar pattern of elemental rings one can observe in whale baleen [69].

The amino acid analyses of the organic material show a rather complicated pattern, with no or very small amounts of both sulfur-containing species (methionine not detected in our and hardly in Sherwood studies) and aromatic amino acids (only thyrosine and phenylalanine detected in small amounts). It is very clearly confirmed in Raman spectra (Figure 5c), where SS (~509 cm^−1^) and CS (~630 cm^−1^) oscillations are either very weak or not at all observed in comparison with the situation for keratins. Contrary to that, the analysis made by electron microprobe confirmed the ordered involvement of S in the ring structure (Figure 3d). The absence of sulfur-containing amino acids testifies that cross-linking cannot be performed by the use of S-S bridges, oppositely as it is in keratins.

### 3.2. Conformation of Peptides

The space organization of the organic material resembles that of keratin. The X-ray diffraction pattern is much similar, with the parts corresponding to the reflections of the coiled matter and sheets. There is one disturbance—the spectrum of the large coral sample is shifted by 2° to the higher values of Θ angles in comparison with all other spectra. One can observe the preponderance of sheet systems over coils in peptides, which more resembles the reptile and bird keratins than the mammalian ones. In addition, the small amount of sulfur-containing amino acids can testify that the amount of amorphous fraction is small [24].

The organic substance comprised in the nodes of bamboo corals is generally called gorgonin. This substance resembles keratins from the total appearance of the X-ray diffraction and Raman spectra. If one compares some subtle details, especially in Raman spectra, the gorgonin seems to be more related to the reptile and bird keratins than to the mammalian analogs. For example, the fragment of the Raman spectrum around amide III, covering the 1140–1390 cm^−1^ range, seems to be relevant for the differentiation of those types of organic materials (Figure 5e). Here, the profile of the large coral spectrum is intermediate between reptile/bird and mammalian keratins. The zone of amide I (Figure 5f) is even best for differentiation. The maxima of the band are put in order: mammalian, bird/reptile, and coralline. The deconvoluted fragment of spectrum in Figure 6 is very similar for all the standards, but bands ascribed to β sheets, both normal and antiparallel, and to random coils, are better visualized in coralline organic matter.

The deconvolution of the amide I band in the Raman spectrum (Figure 6) shows that coral’s gorgonian is much more enriched in β turn structures (zone 8) than the keratins are. Coming back to Table 2, we can observe that amino acids from our measurements attributed to the α helix are on a slightly lower level than in Sherwood studies (except the lysine); those attributed to the β sheets slightly oscillate around Sherwood levels, and those attributed to β turning to prevail in coral material, except aspargin. The results are essentially consistent with the data from Raman measurements.

### 3.3. Cross-Linking

The gorgonin is obviously much less, if at all, cross-linked with the use of sulfur bridges (see SS (~509 cm^−1^)) than popular keratins are; CS (~630 cm^−1^) bands are at the same very low level as reptile and bird keratins but at a lower level than in mammalian keratin (Figure 5c). Thus, the organic sulfur was not detected for cross-linking. Additionally, the aromatic rings (1003 cm^−1^ and 853 cm^−1^) were missing in bamboo corals, contrary to the mammal, bird, and reptile keratins (however, at lower levels in the latter one) (Figure 5d). The aromatic amino acids are known from antipathin, another organic substance similar to gorgonin. The discoveries described in the previous paragraphs contradict the claims about cross-linking via S-S bridges or aromatic amino acids. Perhaps, the dopamine dimer is responsible [55], but it is hardly observed in Figure 5a,b. Dopamine can be synthesized from tyrosine [70], which undoubtedly is present in bamboo corals (Table 2). Ehrlich et al. [53,55] preferred the opinion that the peptide chains were connected through the dicatecholamine (dopamine dimer) links. If so, gorgonin should be a very interesting material for extracting the specifics for nervous system treatments, since it involves the dopamine and bromine compounds. On the other hand, we cannot share the opinion that the heterocyclic systems of pyrrole and indole and imidazole [71] are involved, with their ease of bromination. The bamboo coral samples involve much more bromine than the tissues of any other living organism, but our studies did not allow for establishing the kind of involvement.

In our opinion, the gorgonin in bamboo corals is a kind of analog of keratin-like substances. Those substances arrived in an independent way in the sea environment, while the keratins evolved from reptile/bird type of keratin (mainly β kind) up to mammalian keratins (preferentially in α form).

## 4. Materials and Methods

### 4.1. Materials

Two pieces of skeletons of *Keratoisis* spp. coral colonies from the New Zealand region were provided for this study by the National Institute of Water and Atmospheric Research collection (NIWA,) Samples were collected in the following locations: NIWA Number 16621: (Z9583), 48°2,01′ S, 166°6′ E, Snares Shelf, Campbell Plateau, depth 935 m, small sample; NIWA Number 16,617: (Z9817), 34°48.29′ S, 171°40,17′ E, depth 934 m, large sample.

Both samples were composed of typical node and internode material. The amount of both inorganic and organic material from the sections enabled a wide range of chemical investigations to be carried out.

### 4.2. Methods

Due to the rarity of the samples, we used mainly non-damaging methods (Electron Probe Microanalysis EPMA, X-Ray Diffraction XRD, and Raman Microscopy), but sometimes, it parts of the coral skeleton needed to be dissolved to(amino acid analysis), and then it was necessary to work with the representative samples with mass around several tens of milligrams.

The pieces were cut according to the necessity in the given measurements.

### 4.3. Elementary Analysis

The elementary analyses were performed with the use of an X-ray microanalysis INCA Energy 450 (Oxford Instruments, Abingdon, UK) system with an X-Act Premium detector. The spectral resolution of the detector is 130 eV, as determined for the MnKα line. The microanalytical system was attached to the scanning electron microscope VEGA LMU, produced by Tescan, the Czech Republic. The electron microscope was equipped with a detector of backscattered electrons with YAG crystal, which enabled precise visualization of the studied samples. The working parameters were set as 20 kV (high voltage) and 0.7 nA (beam current), which were optimized for the simultaneous detection of light (mainly forming organic matter) and medium-Z elements, including the main of them—calcium. The elements of interest were identified according to the energy of their K (C, Cl, Na, Mg, S, Ca) and L (Br, I) characteristic X-ray lines. To sharpen the image, enable the elemental measurements, and remove the electric charge from the surface of the sample, the coral material was installed on a layer of conductive glue produced by Agar Scientific company.

### 4.4. Phase Analysis

The phase analysis on a microscale was carried out with the use of a Philips X’Pert MPD diffractometer, with a Cu tube, the photon output of which was collected and guided to the sample through the capillary of 50 μ-wide diameters. The obtained data were identified with the use of the JCPDF (International Centre for Diffraction Data) database by the application of the DIFFRAC plus program for the automatic sorting of spectra. The spectra were collected within an angular range of 5–60 degrees of Θ angles. The lower angular limit was set to detect the X-ray scatterings from the organic matter.

### 4.5. Organic Matter Analysis

The dispersive Raman spectrometer was very useful in identifying organic matter. The inVia Reflex instrument (produced by Renishaw plc, UK) was applied to establish the identity of some chemical entities. As a source, the ion argon laser emitting light of wavelength 514 nm was in use. The initial power of 20 mW was applied. The laser was conventionally air-cooled. The spectral resolution for the laser was <1 cm^−1^. Due to the presence of inorganic and organic components, we decided to study the wide spectral range of 200-3600 cm^−1^. The lens concentrated the laser beam on the sample in 2 μm wide spots. For the control of deposited laser energy on the sample, a set of neutral gray filters was applied. The thermoelectrically cooled CCD detector was used for the collection of spectra. The initial treatment of the data was made using the Wire2 computer program and finished with Origin 8 software. The main Raman bands of interest were as follows: ν (C=O) amide I β-sheet at ~1667 cm^−1^; ν (C=O) amide I α-helix at ~1653 cm^−1^; ν (C-C) in the aromatic ring at 1003 cm^−1^; ν (S-S) at 507 cm^−1^; ν (C-S) at 643 cm^−1^.

## 5. Conclusions

Bamboo corals from a material science point of view are very interesting structures, comprising compact high-magnesium calcite/calcite pieces saturated with apatite inclusions and mixed organic–inorganic nodes. The transverse structure of nodes is ring-like, with an impressive sequence of organic–inorganic layers. The layers are visualized with signals of elements such as P, Br, and S, renormalized against Ca contents, and compared with optical signals. Those signals can potentially be used as palaeochronometers and -thermometers, and up to now, they were poorly exploited. Organic layers are composed of a substance called gorgonin, resembling keratins. The keratins of reptile, bird, and mammalian origin were compared with the gorgonin from the bamboo coral samples. The cross-linking in gorgonin was not supported on S amino acids and single aromatic amino acids. Instead, the cross-linking relying on the dopamine dimer was probably responsible for the compactness of the material. The aromatic compounds and phenylalanine were nearly absent in gorgonin. The gorgonin results resembled somewhat more reptile and bird keratin than that of mammalian origin and can be considered as some independently formed analog of keratins. Generally, the organic substances present in the nodes should be intensively studied, especially those involving bromine. It seems that make up the skeleton’s of bamboo corals in bamboo corals could be seriously considered to have medical applications in the fields of orthopedics, stomatology, and neurology.

## Figures and Tables

**Table 1:** Elemental composition of selected points in bamboo corals, values in ppm.

	Small Coral		Large Coral	
	Ext. Layer	Int. Layer	Ext. Layer	Int. Layer
**Na**	177.0	7.8	13.6	1.2
**Mg**	1109.5	39.3	42.4	5.6
**Fe**	6.5	32.4	14.5	1.8
**Zn**	4.5	16.3	5.3	5.7
**Sr**	161.4	4.0	5.1	0.8
**Br**	4074.0	15,718.0	2814.0	28,847.0
**P**	121.8	153.6	17.9	63.5
**J**	39.8	170.7	56.6	486.0

**Table 2:** Amino acid composition of bamboo corals (μmol/mg) α-helix—red, β-sheet—blue, β-turn—green, other—black.

Amino Acid	Bamboo Coral L., Exterior	Bamboo Coral L., Interior	Bamboo Coral S., Exterior	Bamboo Coral S., Interior	[35] Averaged
Asp	0.346	0.722	0.320	0.609	1.294
Glu	0.332	0.587	0.289	0.593	0.564
Ser	0.439	0.457	0.340	0.337	0.858
Gly	2.911	2.560	3.14	3.201	1.122
His	0.273	0.171	0.158	0.151	
Arg	0.971	0.846	0.930	0.795	0.710
Thr	0.178	0.279	0.434	0.324	0.356
Ala	1.068	0.941	1.048	1.019	1.424
Pro	0.725	0.554	0.544	0.427	
Tyr	0.182	0.117	0.088	0.100	0.164
Val	0.451	0.481	0.570	0.501	0.356
Met					0.052
Ile	0.358	0.376	0.460	0.373	0.226
Leu	0.287	0.240	0.216	0.173	0.214
Phe		0.053	0.138	0.112	0.090
Lys	0.442	0.434	0.410	0.378	

## Data Availability

All collected data were presented in this manuscript.

## References

[1] Armando Sánchez J., Lasker H.R., Taylor D.J. (2003). Phylogenetic analyses among octocorals (*Cnidaria*): Mitochondrial and nuclear DNA sequences (lsu-rRNA, 16S and ssu-rRNA, 18S) support two convergent clades of branching gorgonians. Mol. Phylogenetics Evol..

[2] Smith P.J., McVeagh S.M., Mingoia J.T., France S.C. (2004). Mitochondrial DNA sequence variation in deep-sea bamboo coral (*Keratoisidinae*) species in the southwest and northwest Pacific Ocean. Mar. Biol..

[3] France S.C. (2007). Genetic analysis of *bamboo corals* (*Cnidaria*: *Octocorallia*: *Isididae*): Does lack of colony branching distinguish Lepidisis from Keratoisis?. Bull. Mar. Sci..

[4] Nowak D., Florek M., Nowak J., Kwiatek W., Lekki J., Chevallier P., Hacura A., Wrzalik R., Ben-Nissan B., Van Grieken R. (2009). Morphology and the chemical make-up of the inorganic components of black corals. Mater. Sci. Eng. C.

[5] Clark M.R., Althaus F., Schlacher T.A., Williams A., Bowden D.A., Rowden A.A. (2016). The impacts of deep-sea fisheries on benthic communities: A review. ICES J. Mar. Sci..

[6] Ramirez-Llodra E., Brandt A., Danovaro R., De Mol B., Escobar E., German C.R., Levin L.A., Martinez Arbizu P., Menot L., Buhl-Mortensen P. (2010). Deep, diverse and definitely different: Unique attributes of the world’s largest ecosystem. Biogeosciences.

[7] Shen Y., Guilderson T.P., Sherwood O.A., Castro C.G., Chavez F.P., McCarthy M.D. (2021). Amino acid δ13C and δ15N patterns from sediment trap time series and deep-sea corals: Implications for biogeochemical and ecological reconstructions in paleoarchives. Geochim. Cosmochim. Acta.

[8] Hennige S.J., Wolfram U., Wickes L., Murray F., Roberts J.M., Kamenos N.A., Schofield S., Groetsch A., Spiesz E.M., Aubin-Tam M.E. (2020). Crumbling Reefs and Cold-Water Coral Habitat Loss in a Future Ocean: Evidence of “Coralporosis” as an Indicator of Habitat Integrity. Front. Mar. Sci..

[9] Tracey D.M., Neil H., Marriott P., Andrews A.H., Cailliet G.M., Sanchez J.A. (2007). Age and growth of two genera of deep-sea bamboo corals (*Family isididae*) in New Zealand waters. Bull. Mar. Sci..

[10] Watling L., France S.C. (2021). Toward a Revision of the Bamboo Corals: Part 2, Untangling the Genus Lepidisis (*Octocorallia*: *Isididae*). Bull. Peabody Museum Nat. Hist..

[11] Saucier E.H., France S.C., Watling L. (2021). Toward a revision of the bamboo corals: Part 3, deconstructing the Family Isididae. Zootaxa.

[12] Etnoyer P., Morgan L.E. (2005). Habitat-forming deep-sea corals in the Northeast Pacific Ocean. Cold-Water Corals and Ecosystems.

[13] Bayer F.M., Stefani J. (1987). New and previously known taxa of isidid octocorals (*Coelenterata*: *Gorgonacea*), partly from Antarctic waters. Proc. Biol. Soc. Washingt..

[14] Li J., Wang P. (2019). Discovery of Deep-Water Bamboo Coral Forest in the South China Sea. Sci. Rep..

[15] Hourigan T.F., Lumsden S.E., Dorr G., Bruckner A.W., Brooke S., Stone R.P. (2007). State of deep coral ecosystems of the United States: Introduction and national overview. The State of Deep Coral Ecosystems of the United States.

[16] Etnoyer P.J. Bamboo Corals in North America. Proceedings of the Third International Symposium on Deep-Sea Corals.

[17] Noé S.U., Lembke-Jene L., Dullo W.-C. (2008). Varying growth rates in bamboo corals: Sclerochronology and radiocarbon dating of a mid-Holocene deep-water gorgonian skeleton (*Keratoisis* sp.: *Octocorallia*) from Chatham Rise (New Zealand). Facies.

[18] Bond Z.A. (2005). Development of proxy seawater records from gorgonian coral skeletons. Doctoral Thesis.

[19] Spero H.J., Jang N.A., Adkins J.F. (2003). Geochemistry of the deep water coral Isidella; Intermediate depth and surface ocean chemical recorder. Geochim. Et Cosmochim. Acta Suppl..

[20] Noé S.U., Dullo W.-C. (2006). Skeletal morphogenesis and growth mode of modern and fossil deep-water isidid gorgonians (*Octocorallia*) in the West Pacific (New Zealand and Sea of Okhotsk). Coral Reefs.

[21] Roark E.B., Guilderson T.P., Flood-Page S., Dunbar R.B., Ingram B.L., Fallon S.J., McCulloch M. (2005). Radiocarbon-based ages and growth rates of bamboo corals from the Gulf of Alaska. Geophys. Res. Lett..

[22] Andrews A.H., Cailliet G.M., Kerr L.A., Coale K.H., Lundstrom C., DeVogelaere A.P. (2005). Investigations of age and growth for three deep-sea corals from the Davidson Seamount off central California. Cold-Water Corals and Ecosystems.

[23] Roark E.B., Guilderson T.P., Dunbar R.B., Fallon S.J., Mucciarone D.A. (2009). Extreme longevity in proteinaceous deep-sea corals. Proc. Natl. Acad. Sci. USA.

[24] Wainwright S.A., Biggs W.D., Currey J.D., Gosline J.M. (1982). Mechanical Design in Organisms.

[25] Lewis J.C., Barnowski T.F., Telesnicki G.J. (1992). Characteristics of Carbonates of Gorgonian Axes (*Coelenterata*, *Octocorallia*). Biol. Bull..

[26] Goldberg W.M. (1976). Comparative study of the chemistry and structure of gorgonian and antipatharian coral skeletons. Mar. Biol..

[27] Block R.J., Bolling D. (1939). the Amino Acid Composition of Keratins. J. Biol. Chem..

[28] Lazarus B.S., Chadha C., Velasco-Hogan A., Barbosa J.D.V., Jasiuk I., Meyers M.A. (2021). Engineering with keratin: A functional material and a source of bioinspiration. iScience.

[29] Sugimoto K. (1928). Iodine in Gorgonian Corals. J. Biol. Chem..

[30] Kingsley R.J., Corcoran M.L., Krider K.L., Kriechbaum K.L. (2001). Thyroxine and vitamin D in the gorgonian Leptogorgia virgulata. Comp. Biochem. Physiol. Part A Mol. Integr. Physiol..

[31] Peng J., Li J., Hamann M.T. (2005). The Marine Bromotyrosine Derivatives. Alkaloids Chem. Biol..

[32] McCall A.S., Cummings C.F., Bhave G., Vanacore R., Page-McCaw A., Hudson B.G. (2014). Bromine Is an Essential Trace Element for Assembly of Collagen IV Scaffolds in Tissue Development and Architecture. Cell.

[33] Oluwabusola E.T., Tabudravu J.N., Al Maqbali K.S., Annang F., Pérez-Moreno G., Reyes F., Jaspars M. (2020). Antiparasitic Activity of Bromotyrosine Alkaloids and New Analogues Isolated from the Fijian Marine Sponge Aplysinella rhax. Chem. Biodivers..

[34] Swatschek D., Schatton W., Kellermann J., Müller W.E., Kreuter J. (2002). Marine sponge collagen: Isolation, characterization and effects on the skin parameters surface-pH, moisture and sebum. Eur. J. Pharm. Biopharm..

[35] Sherwood O.A., Scott D.B., Risk M.J. (2006). Late Holocene radiocarbon and aspartic acid racemization dating of deep-sea octocorals. Geochim. Cosmochim. Acta.

[36] Goldberg W.M. (1978). Chemical changes accompanying maturation of the connective tissue skeletons of gorgonian and antipatharian corals. Mar. Biol..

[37] Thresher R.E., Wilson N.C., MacRae C.M., Neil H. (2010). Temperature effects on the calcite skeletal composition of deep-water gorgonians (*Isididae*). Geochim. Cosmochim. Acta.

[38] Saenger C., Gabitov R.I., Farmer J., Watkins J.M., Stone R. (2017). Linear correlations in bamboo coral δ^13^ C and δ^18^ O sampled by SIMS and micromill: Evaluating paleoceanographic potential and biomineralization mechanisms using δ^11^ B and ∆_47_ composition. Chem. Geol..

[39] Wynn P.M., Fairchild I.J., Borsato A., Spötl C., Hartland A., Baker A., Frisia S., Baldini J.U.L. (2018). Sulphate partitioning into calcite: Experimental verification of pH control and application to seasonality in speleothems. Geochim. Cosmochim. Acta.

[40] Okumura T., Kim H., Kim J., Kogure T. (2018). Sulfate-containing calcite: Crystallographic characterization of natural and synthetic materials. Eur. J. Mineral..

[41] Thresher R. (2009). Environmental and compositional correlates of growth rate in deep-water bamboo corals (*Gorgonacea*; *Isididae*). Mar. Ecol. Prog. Ser..

[42] Weinbauer M.G., Vellmirov B. (1995). Calcium, magnesium and strontium concentrations in the calcite sclerites of Mediterranean gorgonians (*Coelenterata*: *Octocorallia*). Estuar. Coast. Shelf Sci..

[43] Heikoop J.M., Hickmott D.D., Risk M.J., Shearer C.K., Atudorei V. (2002). Potential climate signals from the deep-sea gorgonian coral Primnoa resedaeformis. Hydrobiologia.

[44] Hill T.M., LaVigne M., Spero H.J., Guilderson T., Gaylord B., Clague D. (2012). Variations in seawater Sr/Ca recorded in deep-sea bamboo corals. Paleoceanography.

[45] Flöter S., Fietzke J., Gutjahr M., Nehrke G., Eisenhauer A. (2022). Incorporation of Na and S in bamboo coral skeletons. Chem. Geol..

[46] Thresher R.E., MacRae C.M., Wilson N.C., Fallon S. (2009). Feasibility of age determination of deep-water bamboo corals (*Gorgonacea*; *Isididae*) from annual cycles in skeletal composition. Deep Sea Res. Part I Oceanogr. Res. Pap..

[47] De Carlo T.M., Cohen A.L. (2017). Dissepiments, density bands and signatures of thermal stress in Porites skeletons. Coral Reefs.

[48] Hernandez-Leon S. (2002). Lunar cycle of zooplankton biomass in subtropical waters: Biogeochemical implications. J. Plankton Res..

[49] Risk M.J., Heikoop J.M., Snow M.G., Beukens R. (2002). Lifespans and growth patterns of two deep-sea corals: Primnoa resedaeformis and Desmophyllum cristagalli. Hydrobiologia.

[50] Sherwood O., Thresher R., Fallon S., Davies D., Trull T. (2009). Multi-century time-series of 15N and 14C in bamboo corals from deep Tasmanian seamounts: Evidence for stable oceanographic conditions. Mar. Ecol. Prog. Ser..

[51] Prouty N.G. (2015). Age, Growth Rates, and Paleoclimate Studies of Deep Sea Corals. The State of Deep-Sea Coral and Sponge Ecosystems of the United States Report.

[52] Robinson L.F., Adkins J.F., Frank N., Gagnon A.C., Prouty N.G., Brendan Roark E., de Flierdt T. (2014). van The geochemistry of deep-sea coral skeletons: A review of vital effects and applications for palaeoceanography. Deep Sea Res. Part II Top. Stud. Oceanogr..

[53] Ehrlich H., Etnoyer P., Litvinov S.D., Olennikova M.M., Domaschke H., Hanke T., Born R., Meissner H., Worch H. (2006). Biomaterial structure in deep-sea bamboo coral (*Anthozoa*: *Gorgonacea*: *Isididae*): Perspectives for the development of bone implants and templates for tissue engineering. Materwiss. Werksttech..

[54] Kuczumow A., Gorzelak M., Kosiński J., Lasota A., Blicharski T., Gągała J., Nowak J., Jarzębski M., Jabłoński M. (2022). Hierarchy of Bioapatites. Int. J. Mol. Sci..

[55] Ehrlich H. (2019). Phenomenon of Interspace Mineralization in the Bilayered Organic Matrix of Deep-Sea Bamboo Coral (Anthozoa: Gorgonacea: Isididae). Marine Biological Materials of Invertebrate Origin (Ehrlich H.).

[56] Kreplak L., Doucet J., Dumas P., Briki F. (2004). New Aspects of the α-Helix to β-Sheet Transition in Stretched Hard α-Keratin Fibers. Biophys. J..

[57] Rizzo N., Gardner K., Walls D., Keiper-Hrynko N., Ganzke T., Hallahan D. (2006). Characterization of the structure and composition of gecko adhesive setae. J. R. Soc. Interface.

[58] Akhtar W., Edwards H.G.M. (1997). Fourier-transform Raman spectroscopy of mammalian and avian keratotic biopolymers. Spectrochim. Acta Part A Mol. Biomol. Spectrosc..

[59] Edwards H.G., Hunt D., Sibley M. (1998). FT-Raman spectroscopic study of keratotic materials: Horn, hoof and tortoiseshell. Spectrochim. Acta Part A Mol. Biomol. Spectrosc..

[60] Greaves S.J., Griffith W.P. (1991). Vibrational spectra of catechol, catechol-d2 and -d6 and the catecholate monoanion. Spectrochim. Acta Part A Mol. Spectrosc..

[61] Skieresz-Szewczyk K., Jackowiak H., Buchwald T., Szybowicz M. (2017). Localization of Alpha-Keratin and Beta-Keratin (Corneous Beta Protein) in the Epithelium on the Ventral Surface of the Lingual Apex and Its Lingual Nail in the Domestic Goose (Anser Anser f. domestica) by Using Immunohistochemistry and Raman Microspectros. Anat. Rec..

[62] Kuczumow A., Blicharski T., Gorzelak M., Kosiński J., Lasota A., Gągała J., Nowak J., Jarzębski M., Jabłoński M. (2022). Measurements of Energetic States Resulting from Ion Exchanges in the Isomorphic Crystals of Apatites and Bioapatites. Molecules.

[63] Sherwood O.A., Heikoop J.M., Sinclair D.J., Scott D.B., Risk M.J., Shearer C., Azetsu-Scott K. (2005). Skeletal Mg/Ca in Primnoa resedaeformis: Relationship to temperature?. Cold-Water Corals and Ecosystems.

[64] Kuczumow A., Genty D., Chevallier P., Nowak J., Florek M., Buczyńska A. (2005). X-ray and electron microprobe investigation of the speleothems from Godarville tunnel. X-Ray Spectrom..

[65] Kunze K., Niemann H., Ueberlein S., Schulze R., Ehrlich H., Brunner E., Proksch P., Pée K.-H. (2013). Brominated Skeletal Components of the Marine Demosponges, Aplysina cavernicola and Ianthella basta: Analytical and Biochemical Investigations. Mar. Drugs.

[66] Ramalingam S., Periandy S. (2011). Spectroscopic investigation, computed IR intensity, Raman activity and vibrational frequency analysis on 3-bromoanisole using HF and DFT (LSDA/MPW1PW91) calculations. Spectrochim. Acta Part A Mol. Biomol. Spectrosc..

[67] Mahadevan D., Periandy S., Ramalingam S. (2011). Vibrational spectroscopy (FTIR and FTRaman) investigation using ab initio (HF) and DFT (B3LYP) calculations on the structure of 3-Bromo phenol. Spectrochim. Acta Part A Mol. Biomol. Spectrosc..

[68] Xavier T.S., Rashid N., Hubert Joe I. (2011). Vibrational spectra and DFT study of anticancer active molecule 2-(4-Bromophenyl)-1H-benzimidazole by normal coordinate analysis. Spectrochim. Acta Part A Mol. Biomol. Spectrosc..

[69] Szewciw L.J., de Kerckhove D.G., Grime G.W., Fudge D.S. (2010). Calcification provides mechanical reinforcement to whale baleen α-keratin. Proc. R. Soc. B Biol. Sci..

[70] Dreyer D.R., Miller D.J., Freeman B.D., Paul D.R., Bielawski C.W. (2013). Perspectives on poly(dopamine). Chem. Sci..

[71] Pauletti P.M., Cintra L.S., Braguine C.G., Filho A.A., Silva M.L.A., Cunha W.R., Januário A.H. (2010). Halogenated Indole Alkaloids from Marine Invertebrates. Mar. Drugs.

